# Supplementary figures and images for: A bidirectional switch in the Shank3 phosphorylation state biases synapses toward up- or downscaling
Source: eLife. 2022 Apr 26;11:e74277. doi: 10.7554/eLife.74277 (PMC9084893; doi:10.7554/eLife.74277)

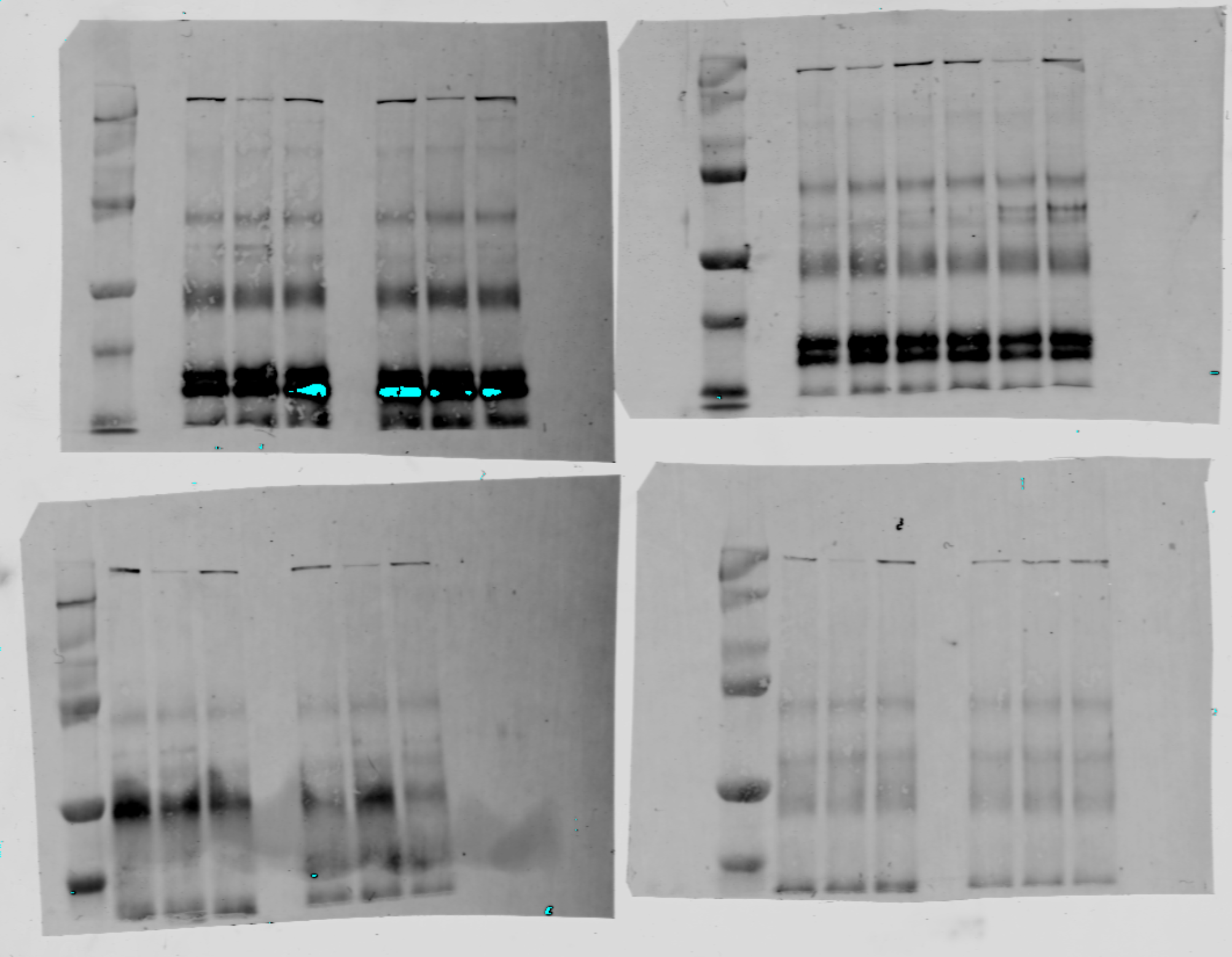

Supplement: Figure 2—source data 2. [file elife-74277-fig2-data2.zip › Figure 2 - source data 2 - blot image/Original blots stained for pS1615.tif]

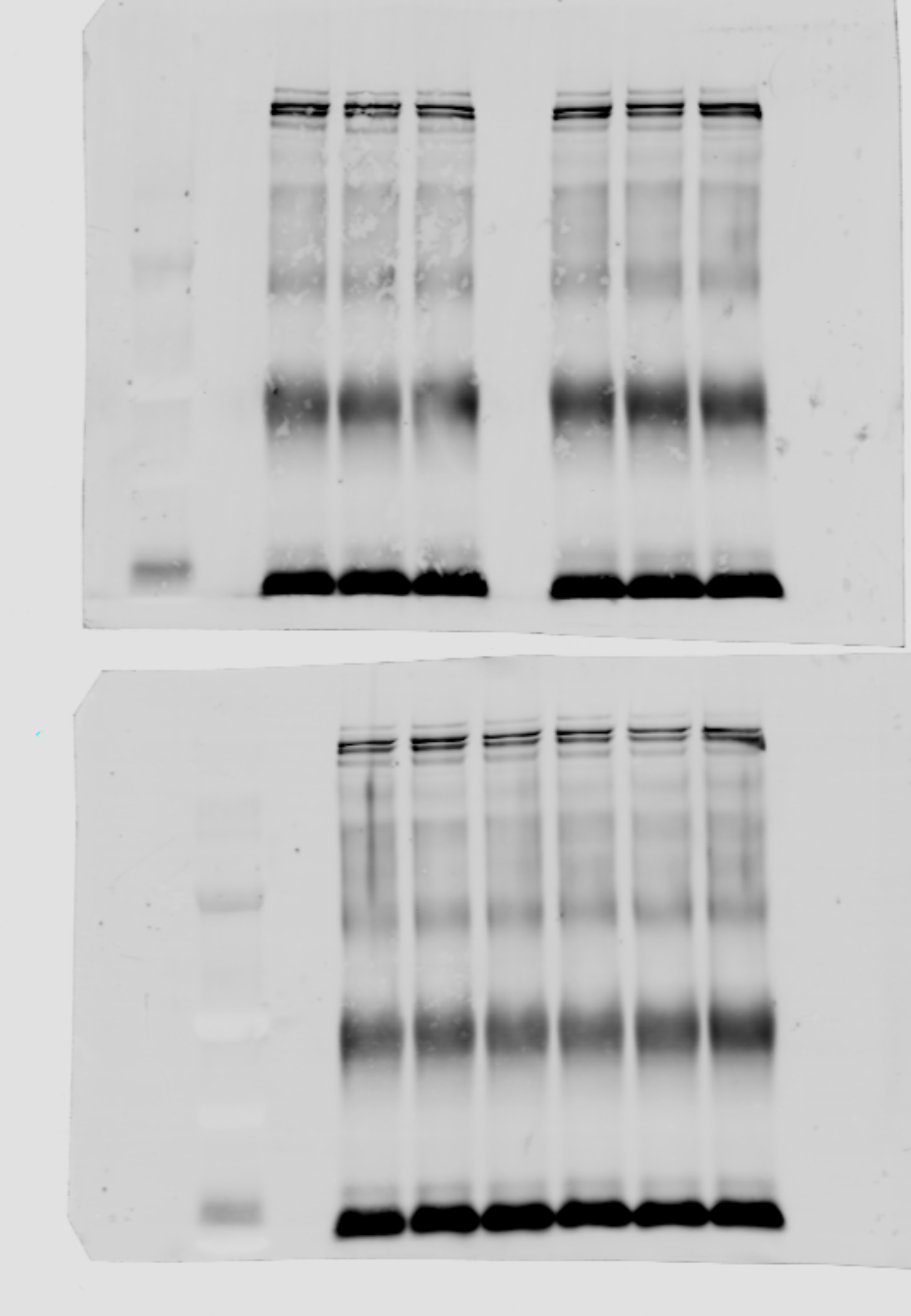

Supplement: Figure 2—source data 2. [file elife-74277-fig2-data2.zip › Figure 2 - source data 2 - blot image/Original blots stained for Shank3.tif]

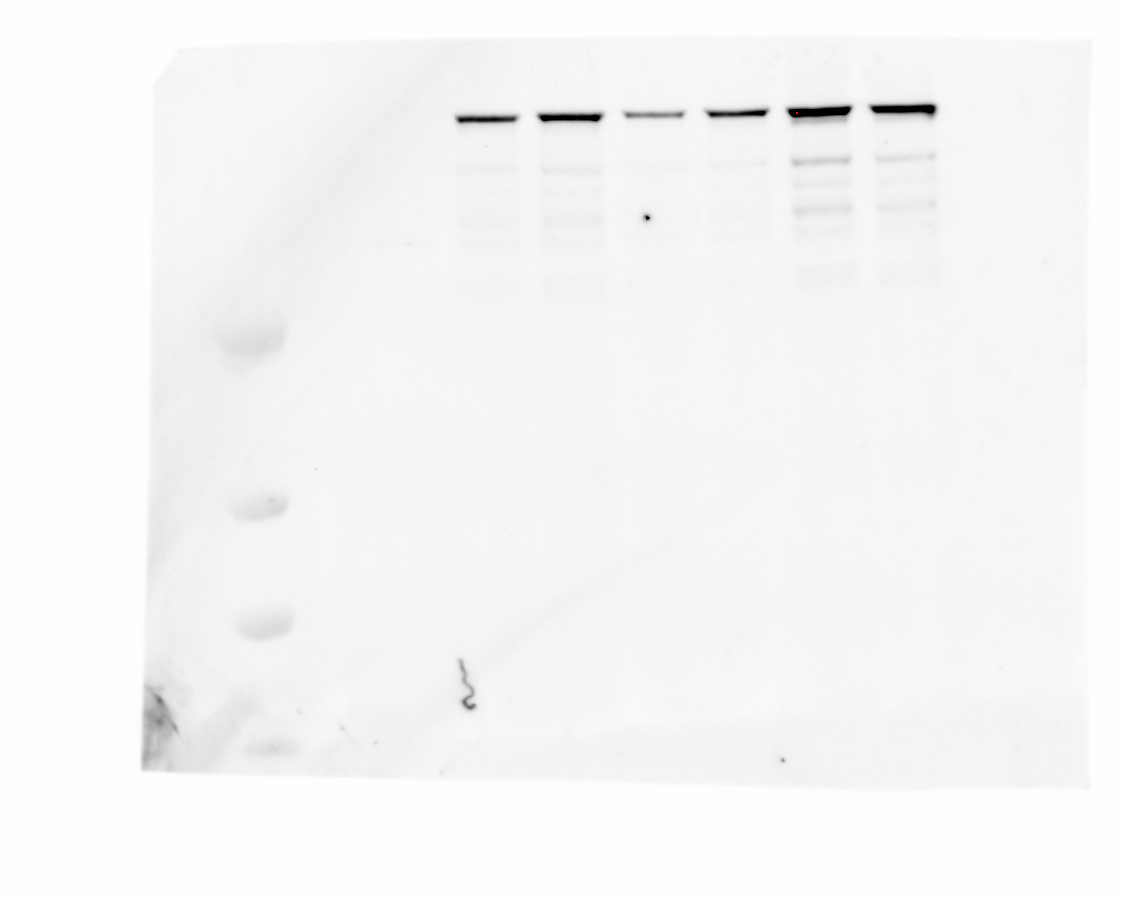

Supplement: Figure 2—figure supplement 1—source data 1. [file elife-74277-fig2-figsupp1-data1.zip › Figure 2 - figure supplement 1 - source data 1 - blot image/Original blot stained for HA in A.tif]

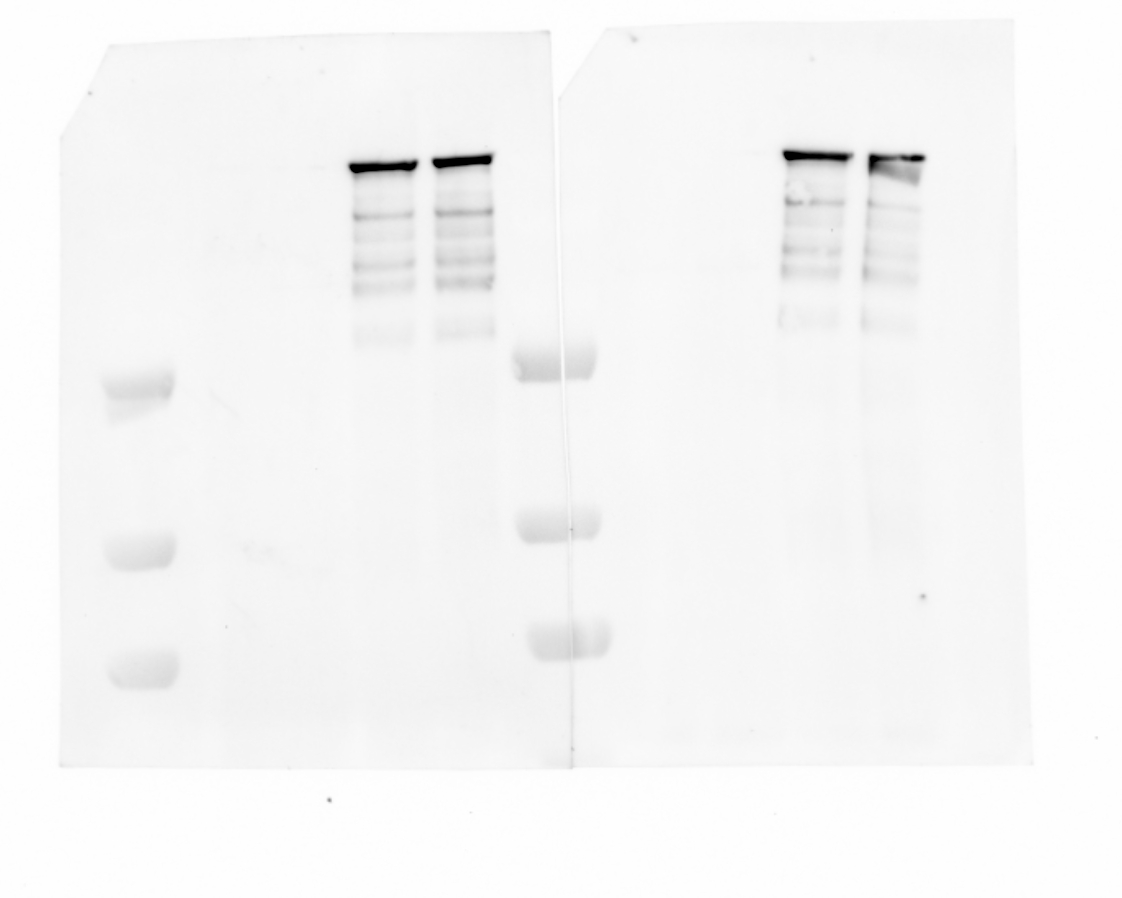

Supplement: Figure 2—figure supplement 1—source data 1. [file elife-74277-fig2-figsupp1-data1.zip › Figure 2 - figure supplement 1 - source data 1 - blot image/Original blot stained for HA in B.jpg]

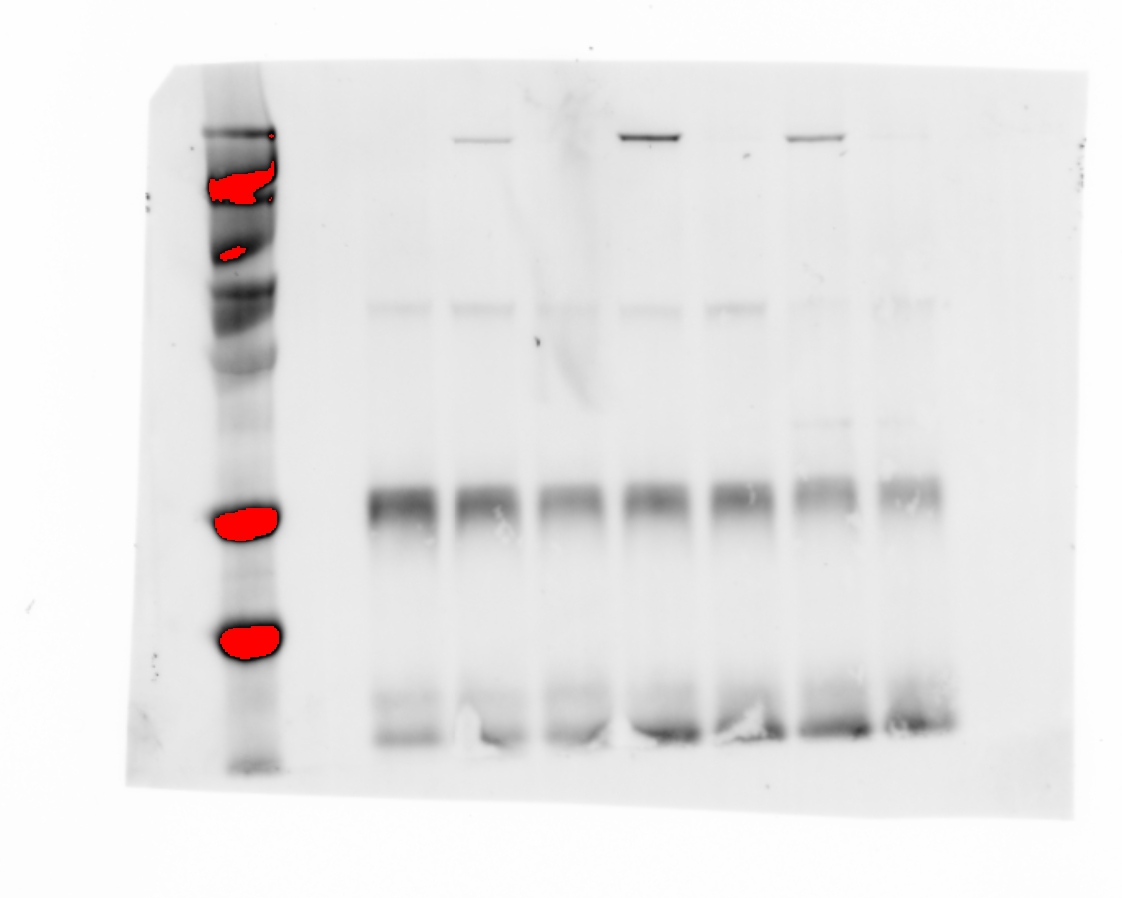

Supplement: Figure 2—figure supplement 1—source data 1. [file elife-74277-fig2-figsupp1-data1.zip › Figure 2 - figure supplement 1 - source data 1 - blot image/Original blot stained for pS1615 in A.jpg]

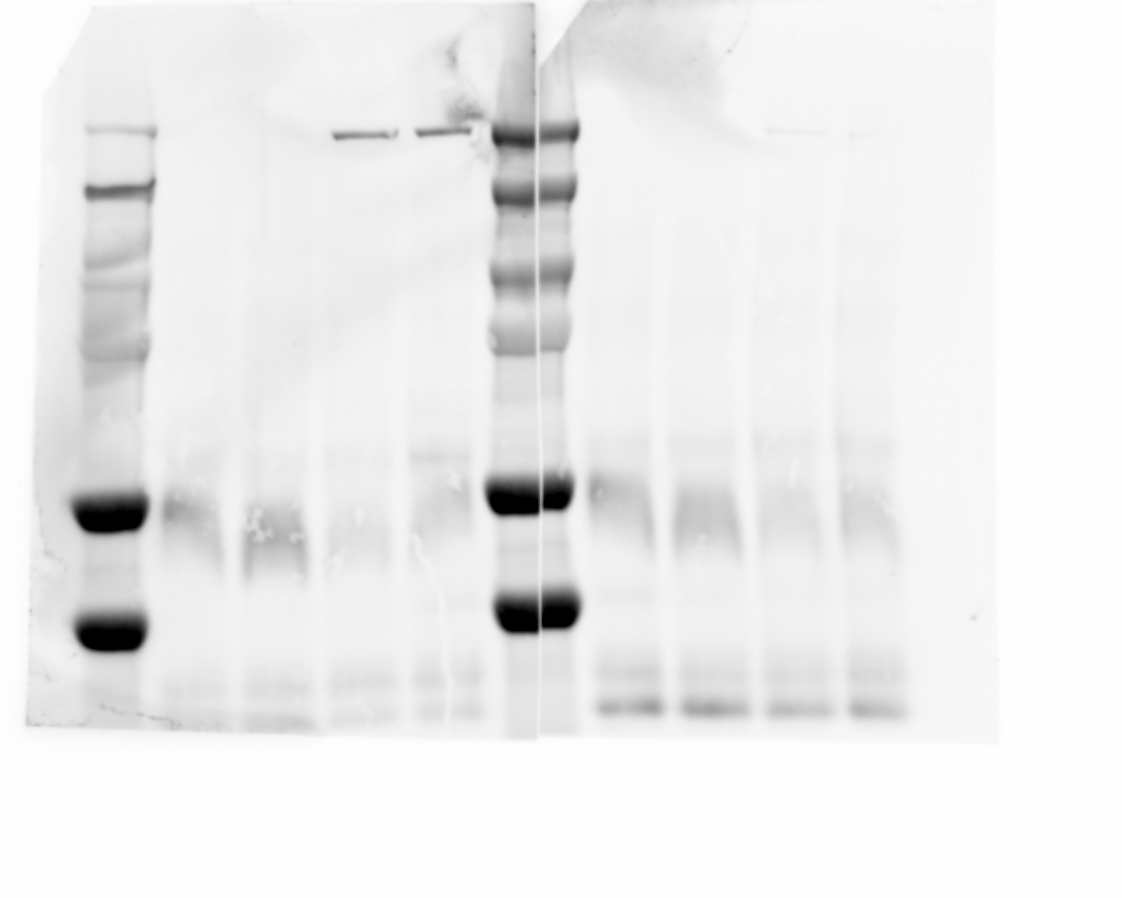

Supplement: Figure 2—figure supplement 1—source data 1. [file elife-74277-fig2-figsupp1-data1.zip › Figure 2 - figure supplement 1 - source data 1 - blot image/Original blot stained for pS1615 in B.tif]

Uncropped Western blots used in  
Figure 2 — figure supplement 1

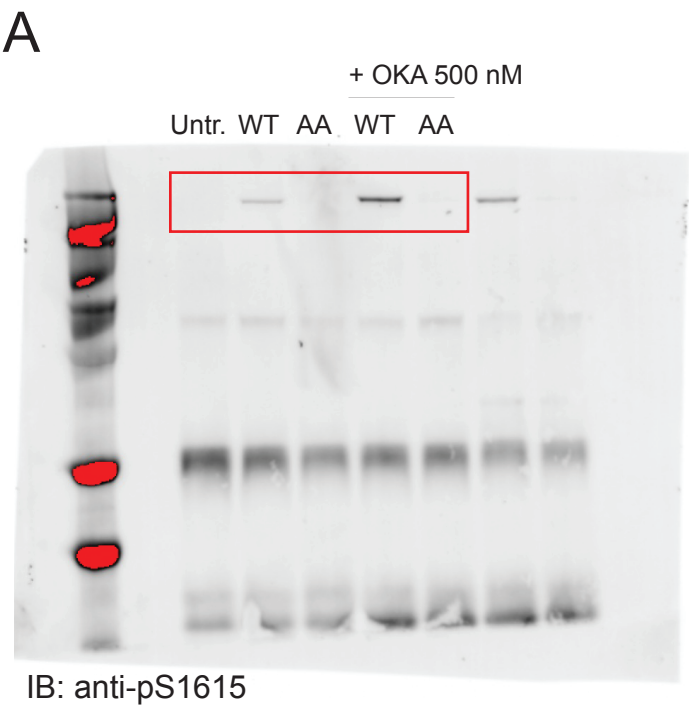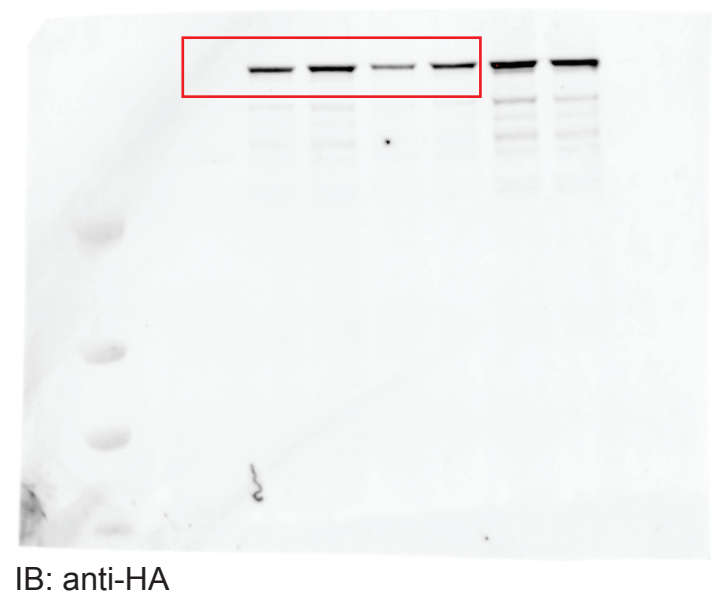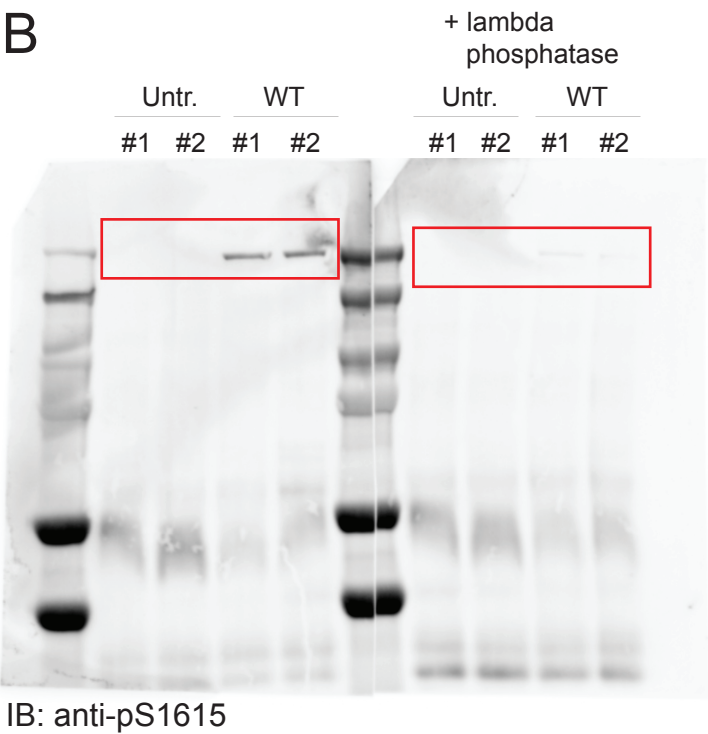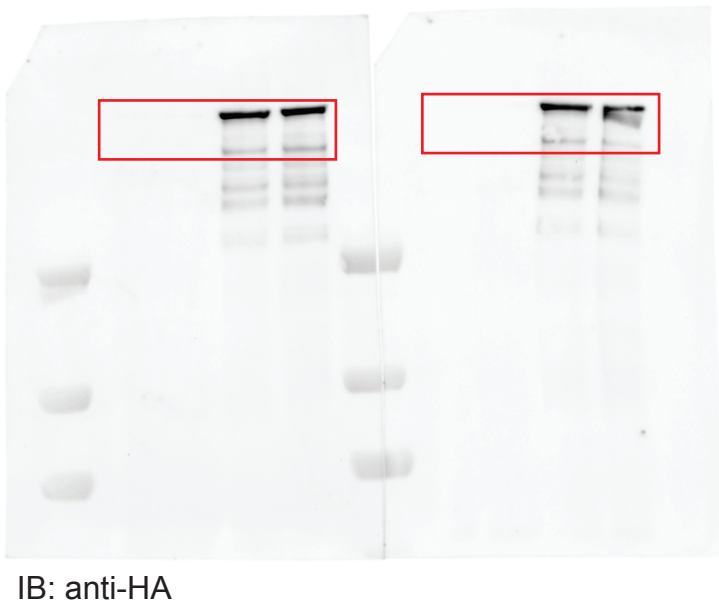

Supplement: Figure 2—figure supplement 1—source data 1. [file elife-74277-fig2-figsupp1-data1.zip › Figure 2 - figure supplement 1 - source data 1 - blot image/Figure 2 - figure supplement 1 - uncropped blots.pdf]

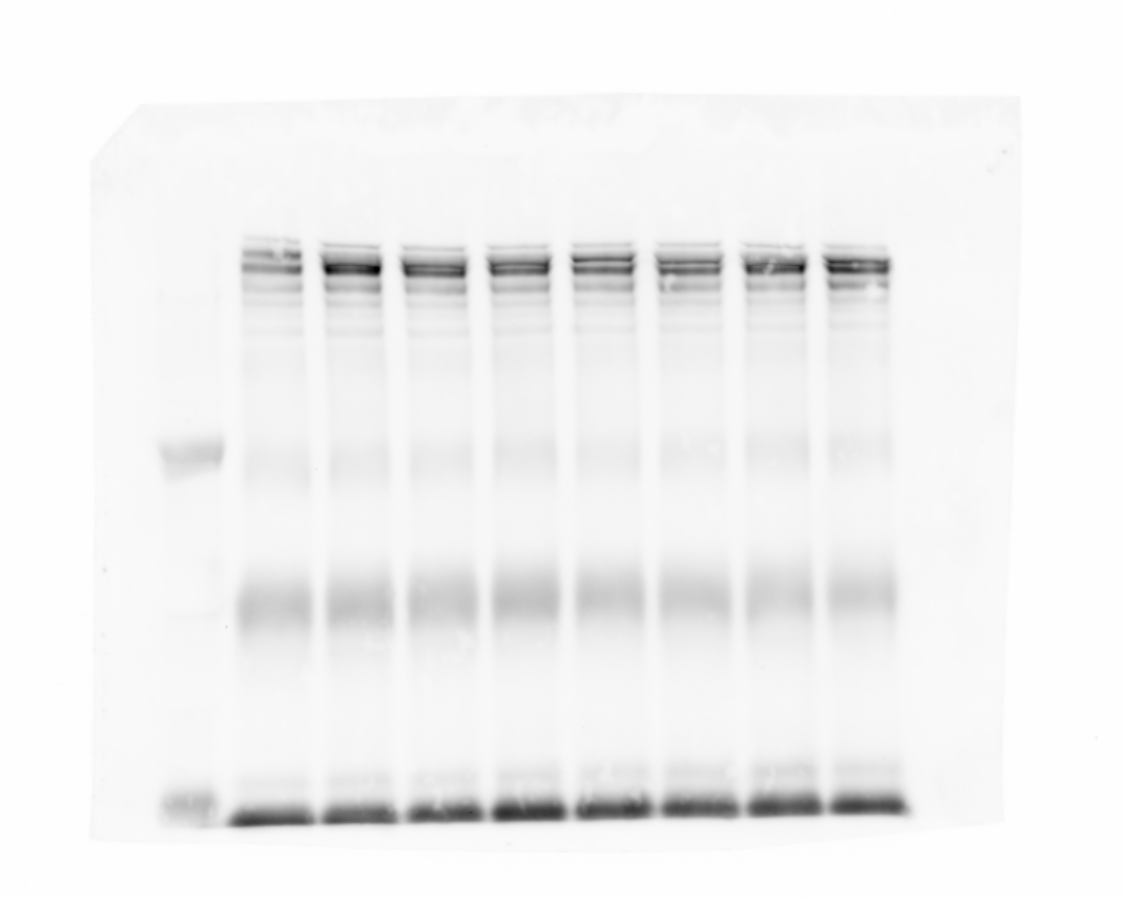

Supplement: Figure 4—source data 2. [file elife-74277-fig4-data2.zip › Figure 4 - source data 2 - blot image/Original blot stained for Shank3 in B.tif]

Uncropped Western blots used in Figure 4B

B

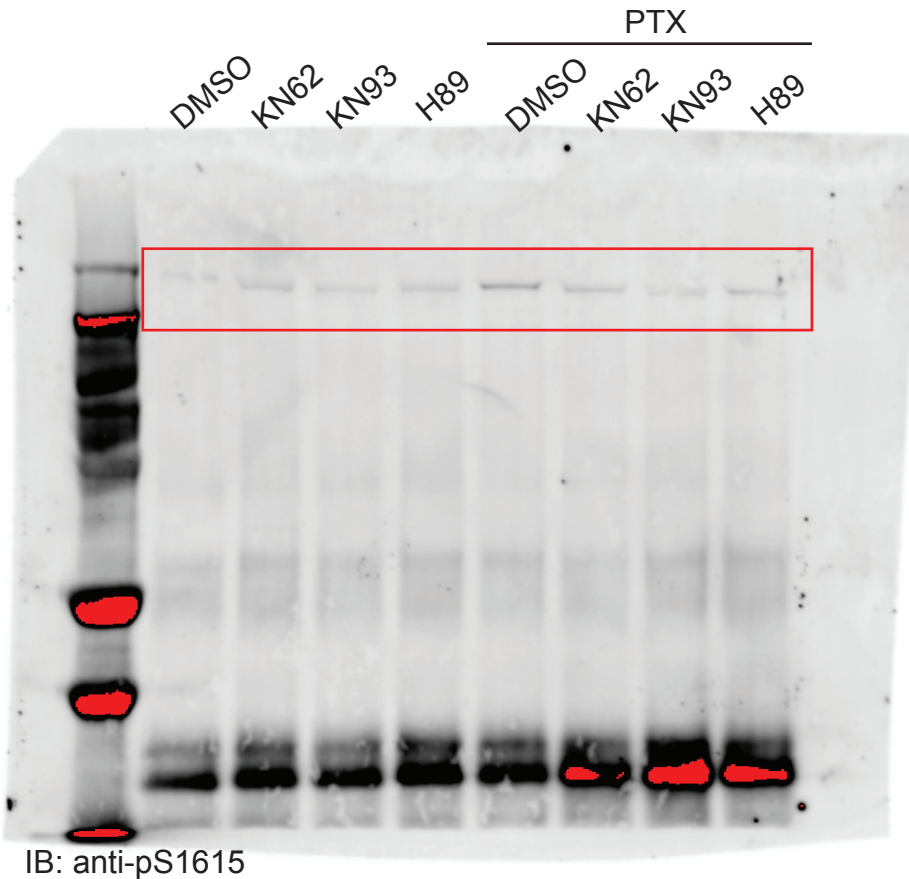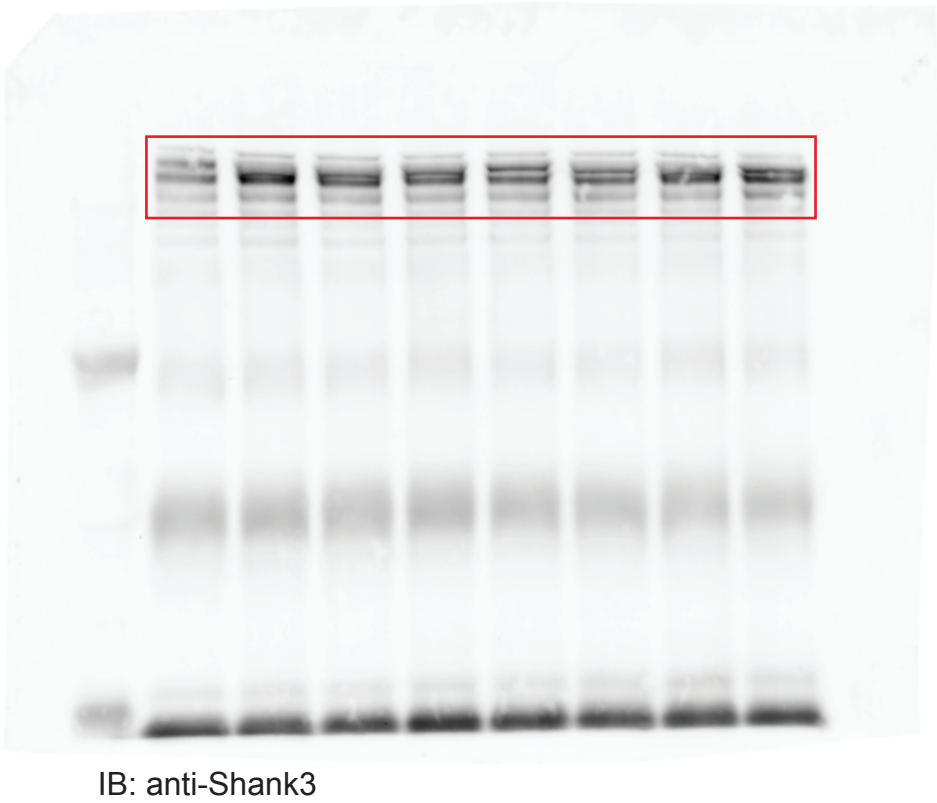

Supplement: Figure 4—source data 2. [file elife-74277-fig4-data2.zip › Figure 4 - source data 2 - blot image/Figure 4B - uncropped blots.pdf]

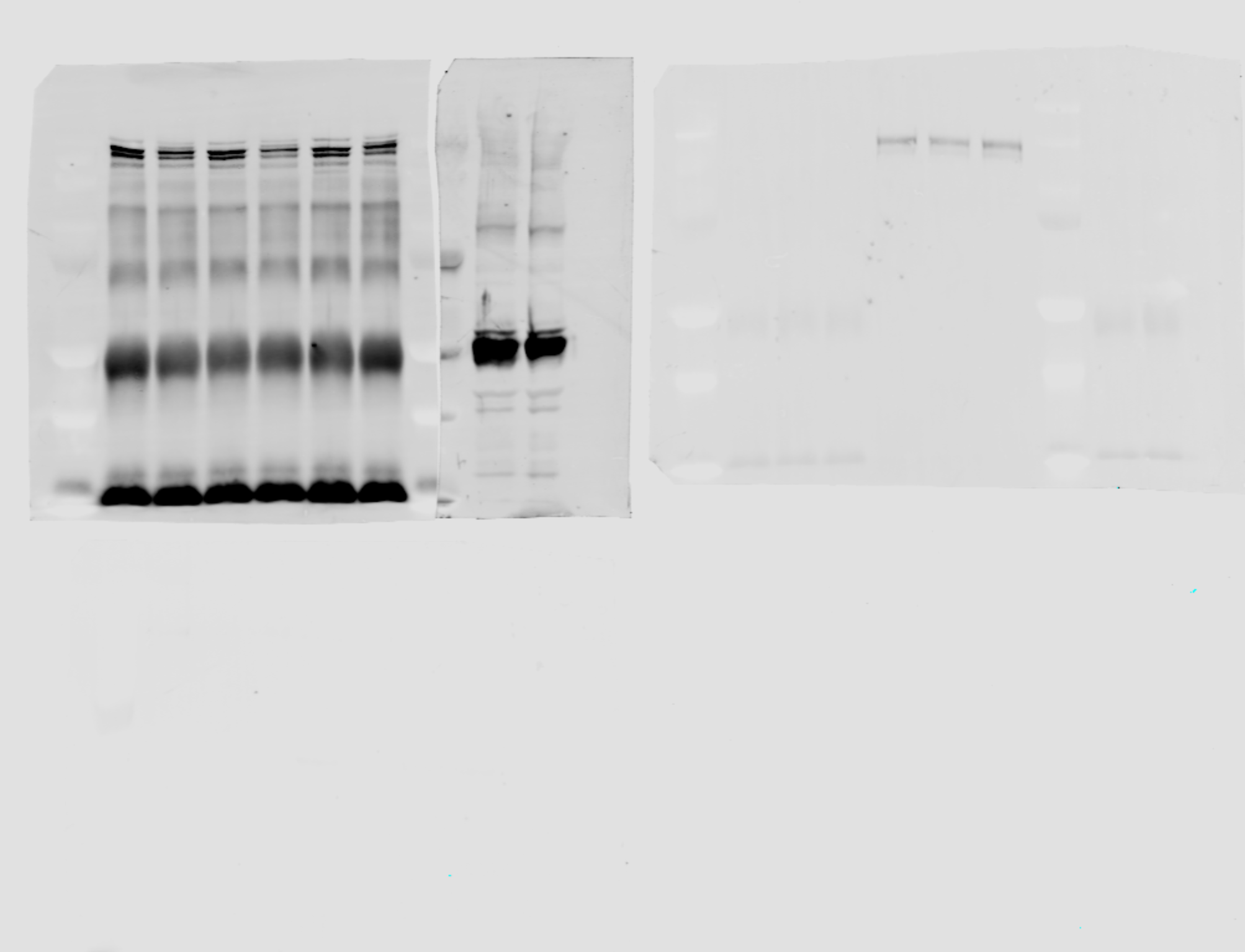

Supplement: Figure 4—source data 2. [file elife-74277-fig4-data2.zip › Figure 4 - source data 2 - blot image/Original blot stained for Shank3 in G.tif]

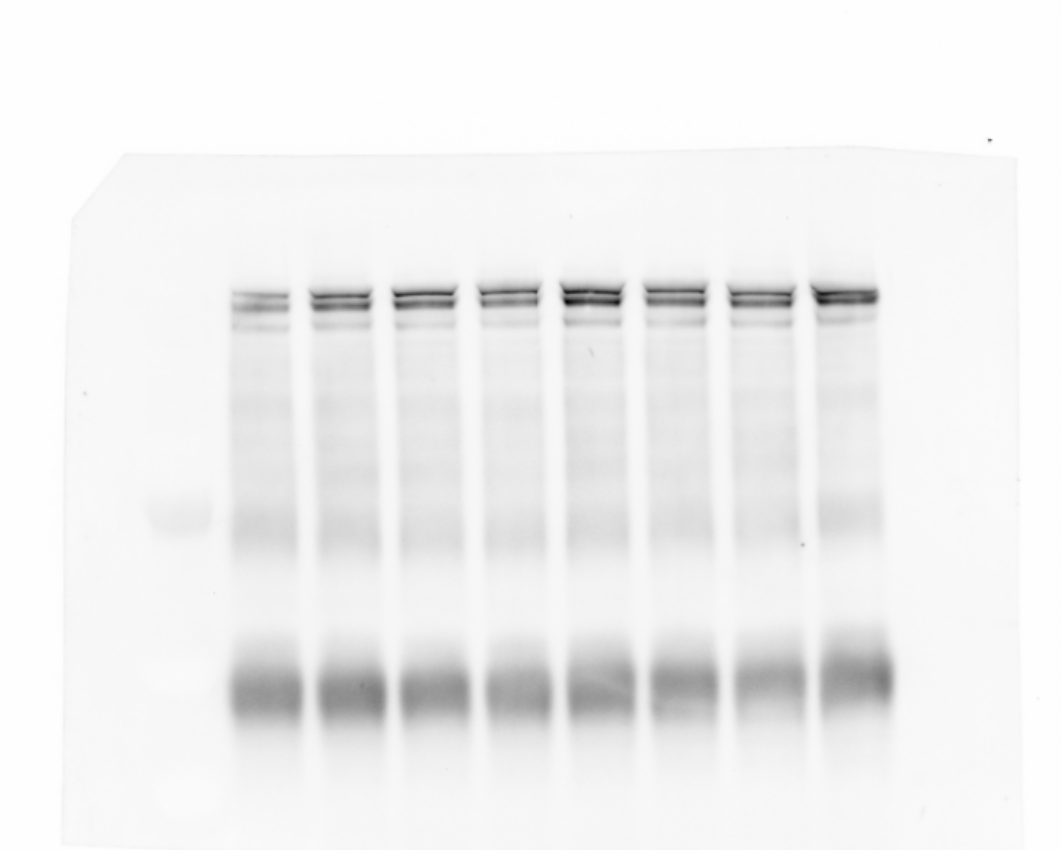

Supplement: Figure 4—source data 2. [file elife-74277-fig4-data2.zip › Figure 4 - source data 2 - blot image/Original blot stained for Shank3 in F.tif]

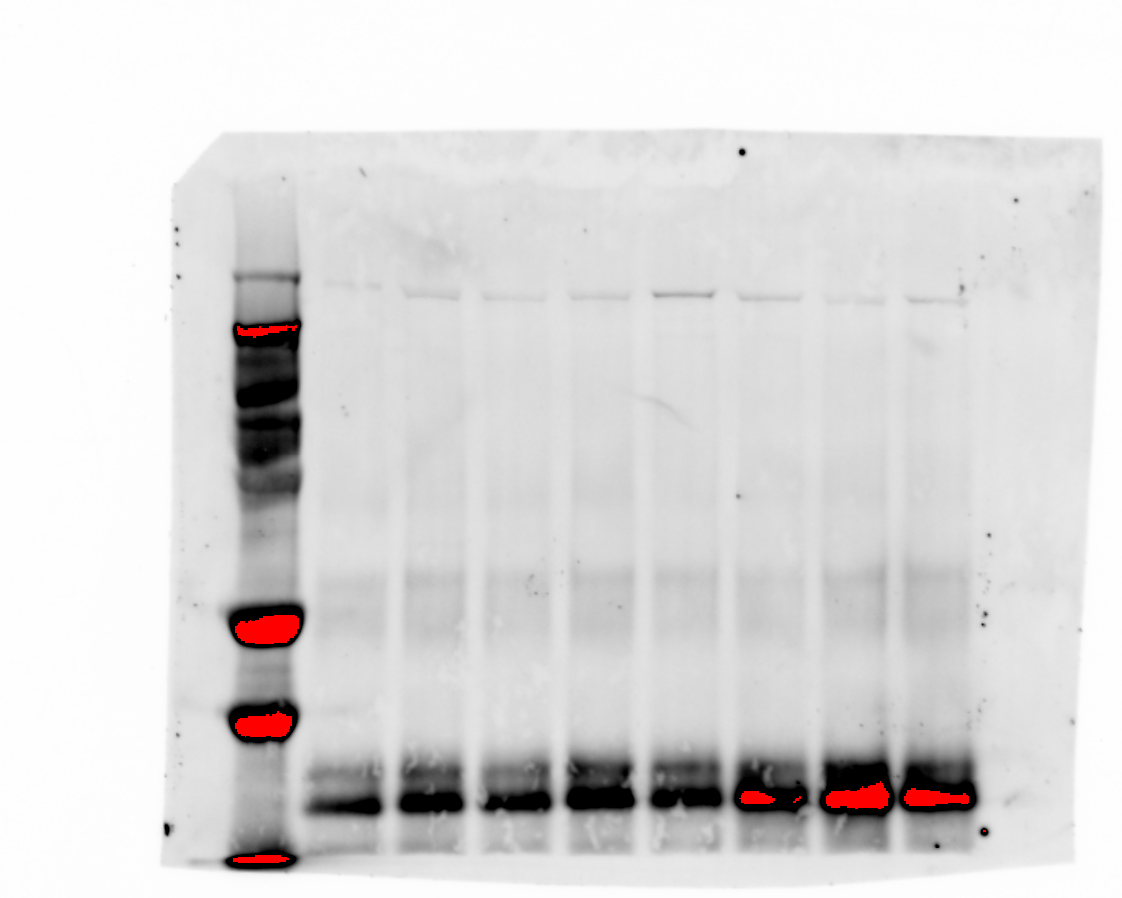

Supplement: Figure 4—source data 2. [file elife-74277-fig4-data2.zip › Figure 4 - source data 2 - blot image/Original blot stained for pS1615 in B.tif]

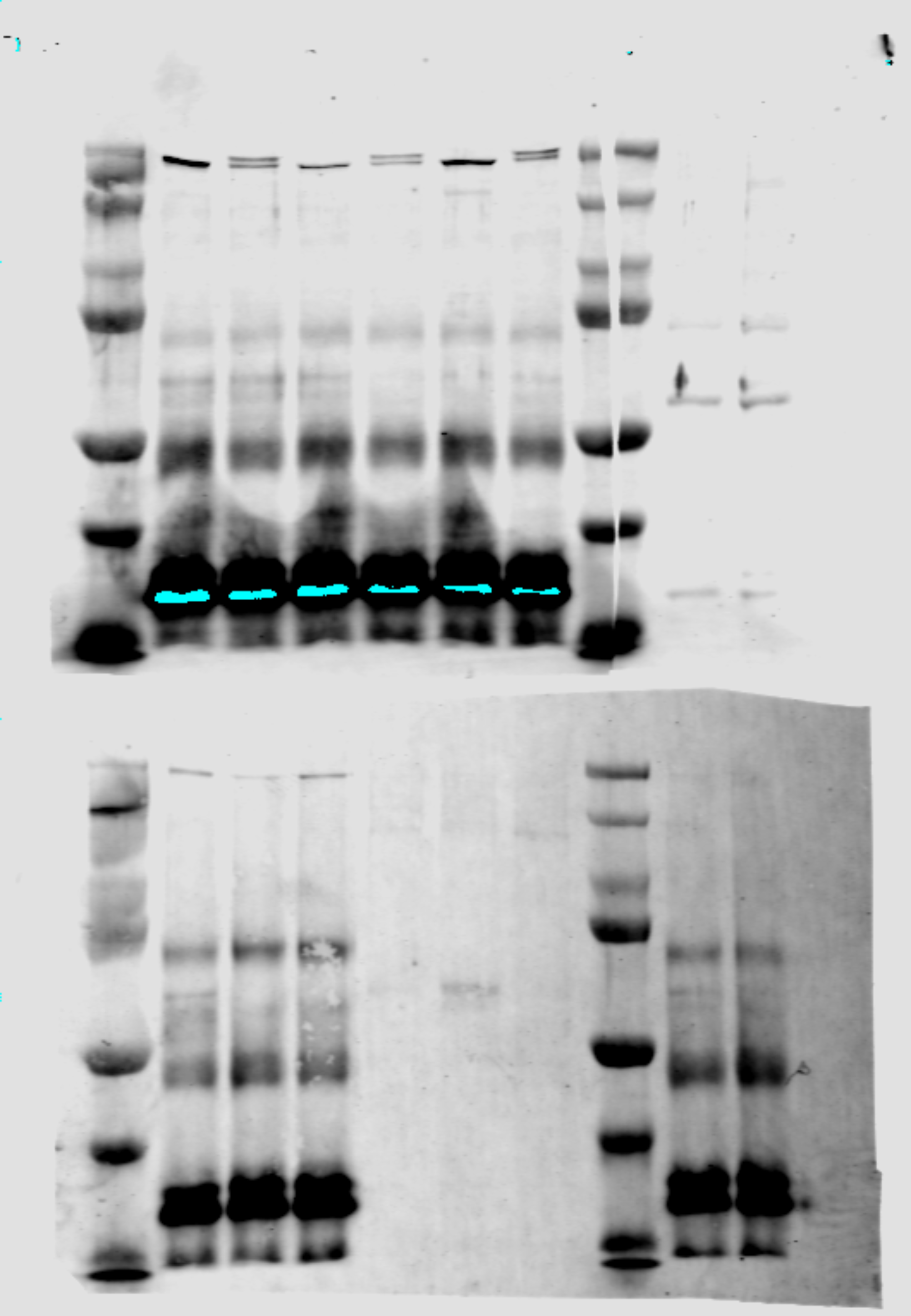

Supplement: Figure 4—source data 2. [file elife-74277-fig4-data2.zip › Figure 4 - source data 2 - blot image/Original blot stained for pS1615 in G.tif]

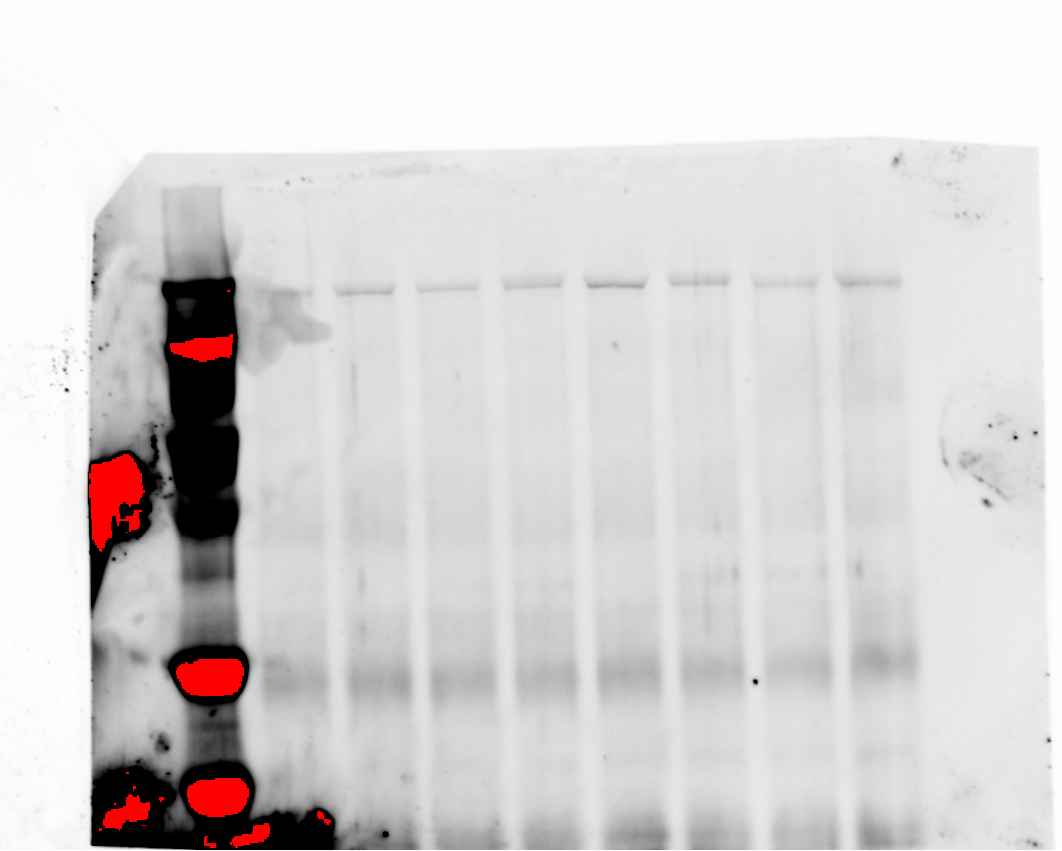

Supplement: Figure 4—source data 2. [file elife-74277-fig4-data2.zip › Figure 4 - source data 2 - blot image/Original blot stained for pS1615 in F.tif]

Uncropped Western blots used in  
Figure 4 — figure supplement 1

A

● ● ● TTX 24 hr  
● ● ● OKA (500 nM)

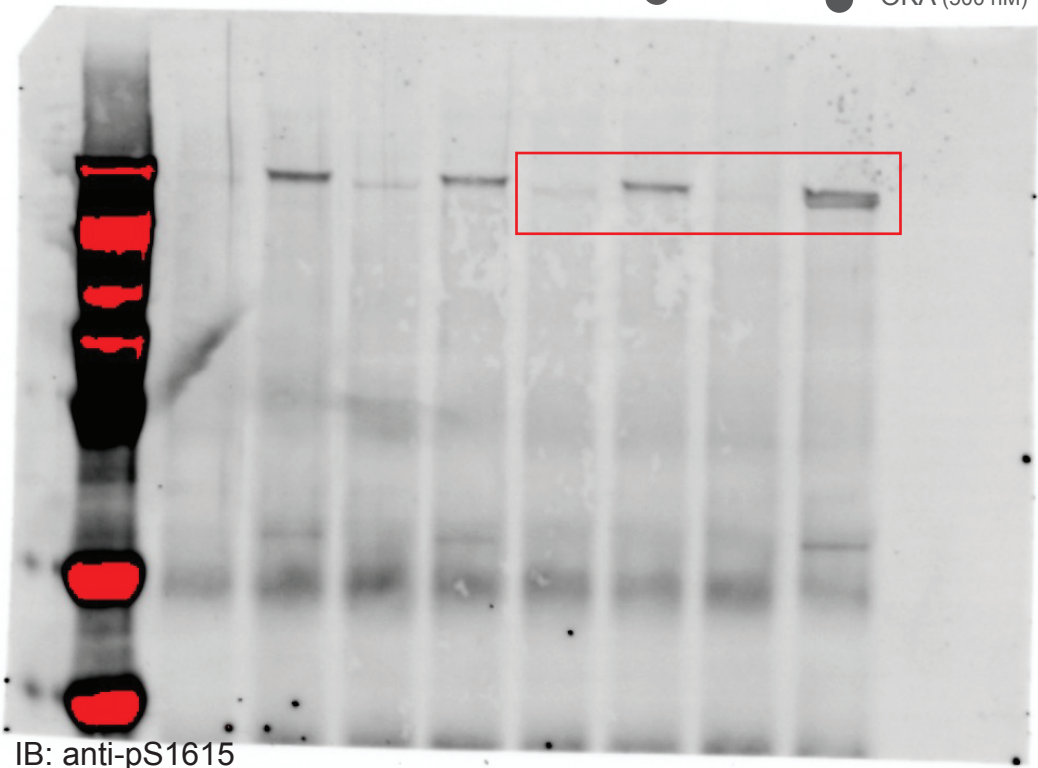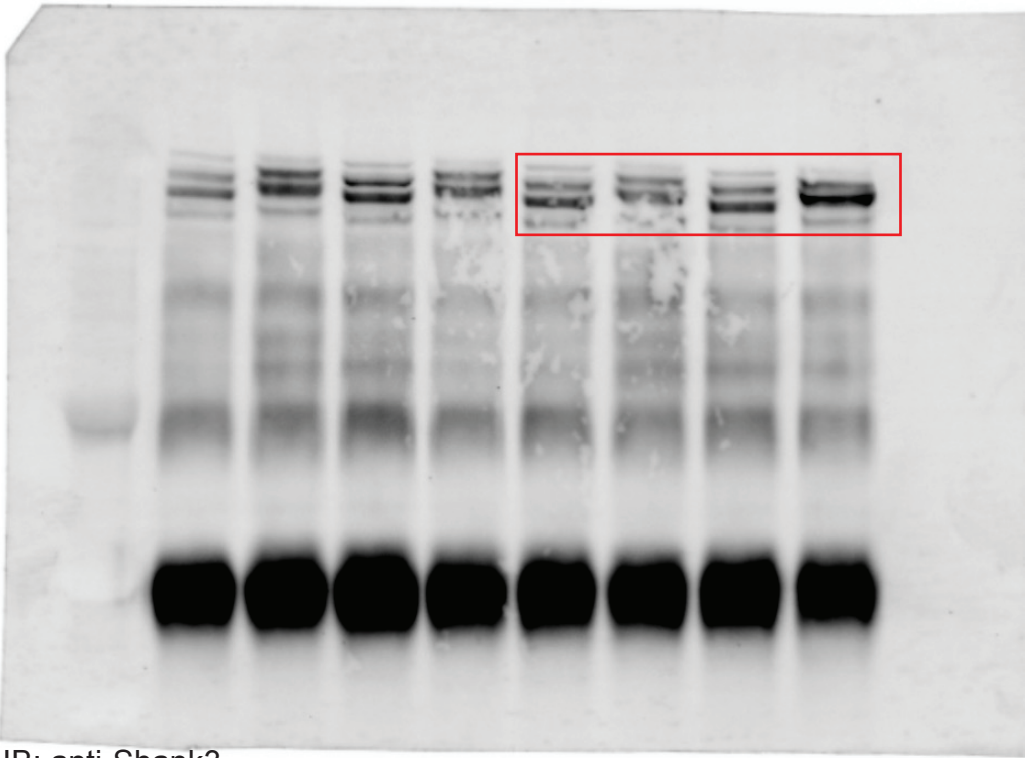

Supplement: Figure 4—figure supplement 1—source data 2. [file elife-74277-fig4-figsupp1-data2.zip › Figure 4 - figure supplement 1 - source data 2 - blot image /Figure 4 - figure supplement 1 - uncropped blots.pdf]

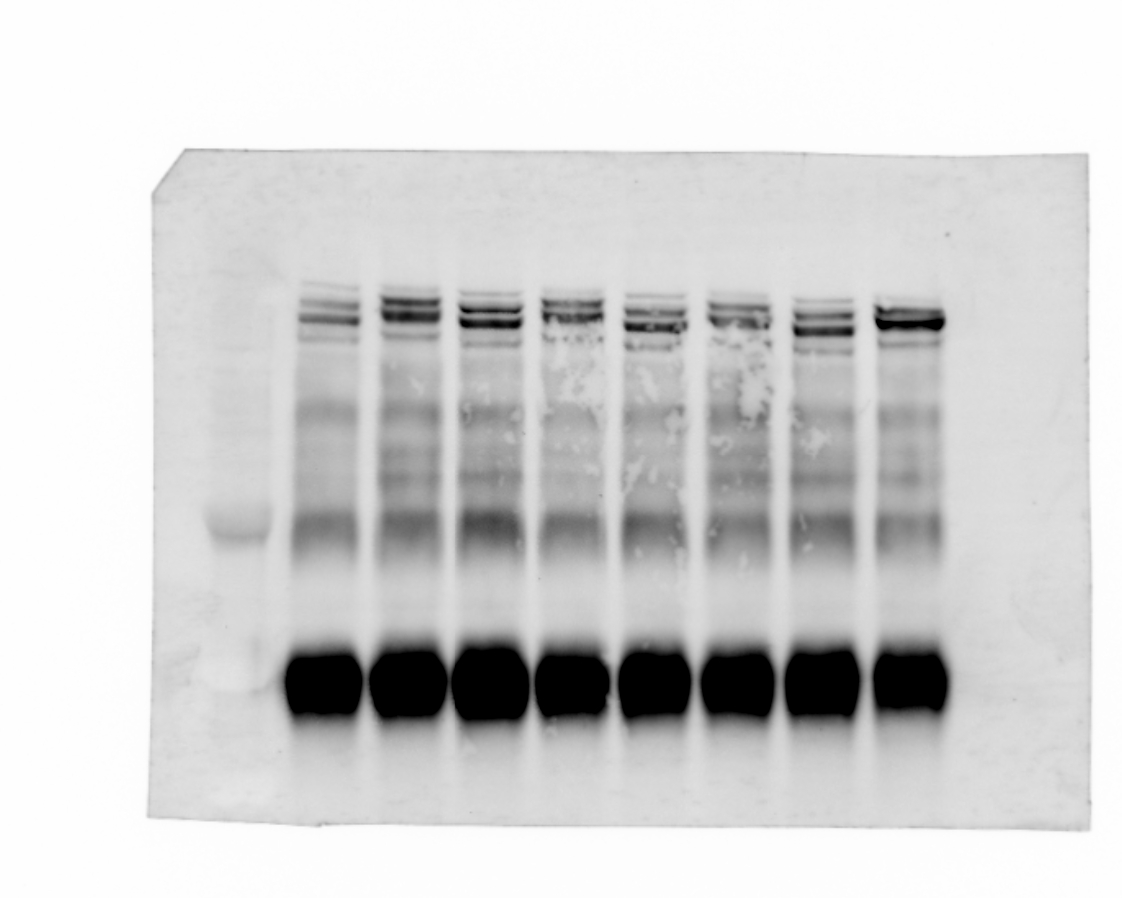

Supplement: Figure 4—figure supplement 1—source data 2. [file elife-74277-fig4-figsupp1-data2.zip › Figure 4 - figure supplement 1 - source data 2 - blot image /Original blot stained for Shank3.jpg]

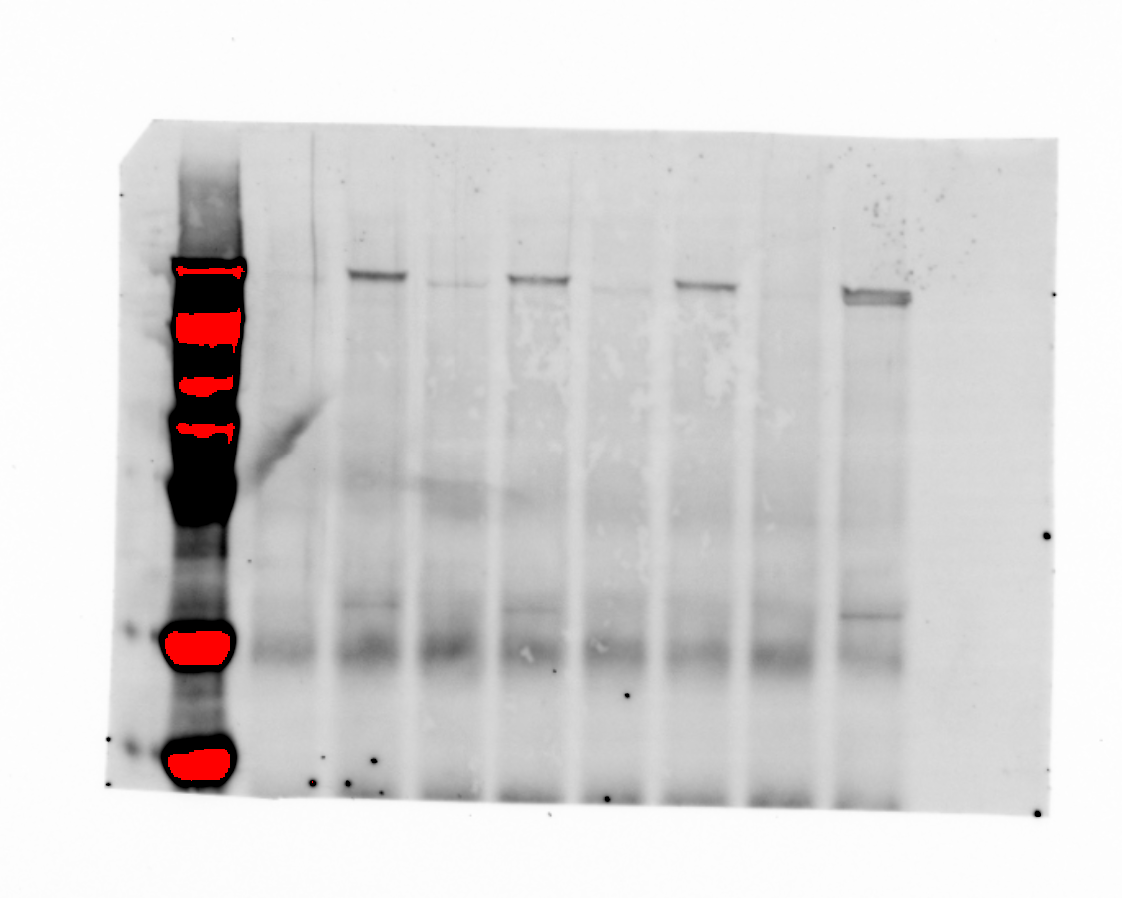

Supplement: Figure 4—figure supplement 1—source data 2. [file elife-74277-fig4-figsupp1-data2.zip › Figure 4 - figure supplement 1 - source data 2 - blot image /Original blot stained for pS1615.tif]
