# Supplementary material for: A bidirectional switch in the Shank3 phosphorylation state biases synapses toward up- or downscaling
Source: eLife. 2022 Apr 26;11:e74277. doi: 10.7554/eLife.74277 (PMC9084893; doi:10.7554/eLife.74277)

Uncropped Western blots used in Figure 4F, G

F

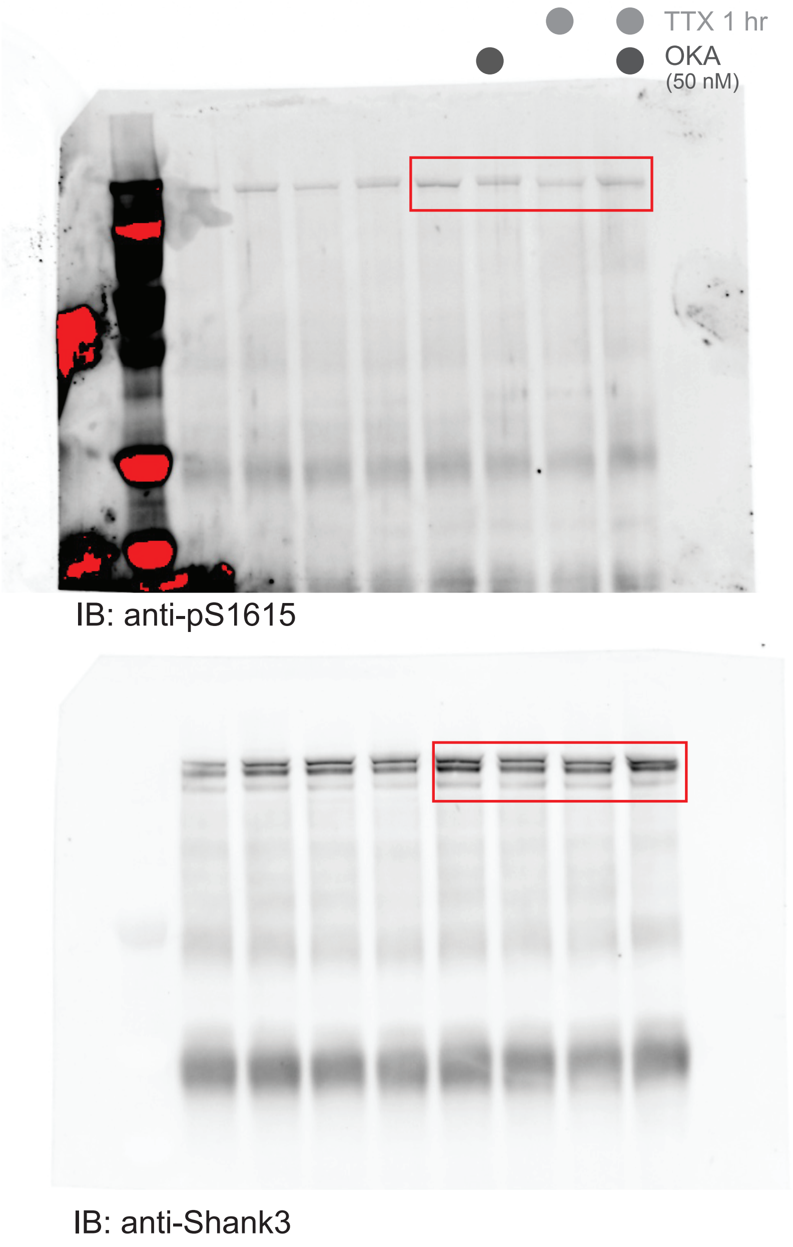

G

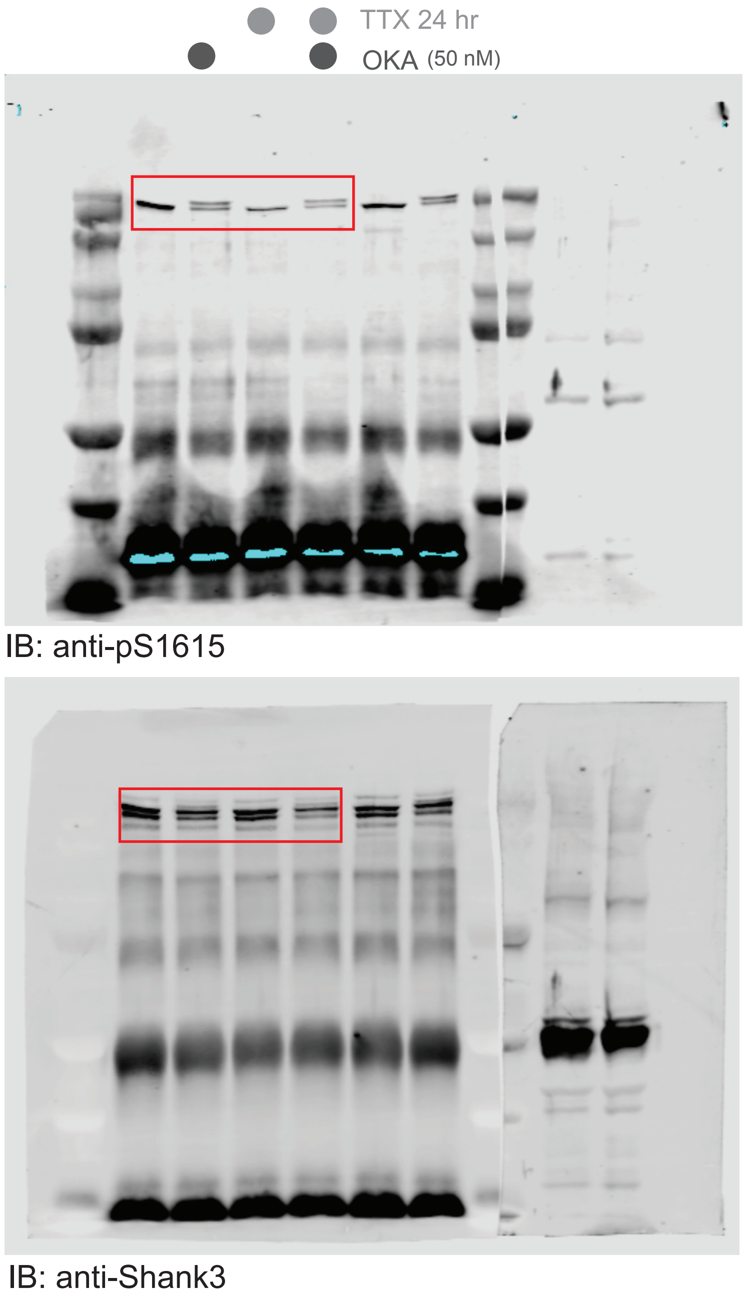

Supplement: Figure 4—source data 2. [file elife-74277-fig4-data2.zip › Figure 4 - source data 2 - blot image/Figure 4FG - uncropped blots.pdf]
